# A deep-dive into fictive locomotion – a strategy to probe cellular activity during speed transitions in fictively swimming zebrafish larvae

**DOI:** 10.1242/bio.059167

**Published:** 2022-03-22

**Authors:** Harmen Kornelis Koning, Aikeremu Ahemaiti, Henrik Boije

**Affiliations:** Department of Immunology, Genetics and Pathology, Uppsala University, S-751 08, Uppsala, Sweden

**Keywords:** Calcium imaging, Locomotor network, Optogenetics, Optomotor response

## Abstract

Fictive locomotion is frequently used to study locomotor output in paralyzed animals. We have evaluated the character of swim episodes elicited by different strategies in zebrafish. Motor output was measured on both sides of a body segment using electrodes and a pipeline for synchronizing stimulation and recording, denoising data and peak-finding was developed. The optomotor response generated swims most equivalent to spontaneous activity, while electrical stimulation and NMDA application caused various artefacts. Our optimal settings, optomotor stimulation using 5-day-old larvae, were combined with calcium imaging and optogenetics to validate the setup's utility. Expression of GCaMP5G by the *mnx1* promoter allowed correlation of calcium traces of dozens of motor neurons to the fictive locomotor output. Activation of motor neurons through channelrhodopsin produced aberrant locomotor episodes. This strategy can be used to investigate novel neuronal populations in a high-throughput manner to reveal their role in shaping motor output.

This article has an associated First Person interview with the first author of the paper.

## Abstract (precis)

**Summary:** This approach combines fictive locomotion, elicited through the optomotor response, and calcium imaging or optogenetics, to investigate the role of neuronal populations in shaping motor output.

## INTRODUCTION

The locomotor network is a commonly used model to study neuronal networks due to its clearly defined components and relatively simplistic, repetitive, output. Our aim was to develop a strategy to study the role of specific neuronal populations during speed transitions.

Fictive locomotion is a benchmark method used in locomotor studies where the spinal cord is dissected out or the entire animal is paralyzed and electrodes are placed on ventral roots or musculature to measure motor neuron output. The resulting fictive motor activity closely resembles that of a freely moving animal with the advantage of an immobile spinal cord that allows motion-sensitive techniques such as fluorescent imaging, optogenetic manipulation, or electrophysiology to be performed. Fictive locomotion has been widely used in mammals (Cazalets et al., 1992; Kriellaars et al., 1994; Meehan et al., 2012), non-mammalian vertebrates (Cohen and Harris-Warrick, 1984; Fetcho and Svoboda, 1993; Masino et al., 2005; Zhang et al., 2009), and invertebrates (Delcomyn, 1980; Marder and Bucher, 2007; Pulver et al., 2015).

A number of studies have characterized fictive motor output of zebrafish larvae during early development, in classical locomotor mutants, and to investigate descending control of locomotion (Buss and Drapeau, 2001; Masino et al., 2005; McLean et al., 2008; Severi et al., 2014). To evoke fictive swimming, different stimuli have been used: change of illumination levels (Buss and Drapeau, 2002), electrical stimulation (Kimura and Higashijima, 2019; Roberts et al., 2019), application of drugs (Coslovich et al., 2018; McDearmid and Drapeau, 2006; Thomas et al., 2009), or a moving visual stimulus to trigger the optomotor response (Kist and Portugues, 2019; Orger et al., 2008). These methods all have their advantages and disadvantages, but no consensus is met on the differences in the locomotor output generated or how this affects the recruitment of neurons within the network. A thorough comparison is thus warranted to elucidate the inherent differences in motor behavior for the different strategies of evoking fictive swims.

The zebrafish locomotor network has a highly modular architecture composed of speed-dependent sub-circuits. In larvae, slow swims, with tail beat frequencies under 30 Hz, are driven by small secondary motor neurons located ventrally in the spinal cord (McLean and Fetcho, 2008). As swim speed increases, a pool of larger, more dorsally located motor neurons are recruited (McLean et al., 2008). The slow and fast motor neurons receive input from distinct pools of fast and slow V2a and V0v interneurons (Bhatt et al., 2007; McLean et al., 2007), forming the separate speed modules*.* In this study, we focused on maximizing the number of swims that cross the threshold of 30 Hz and thus comprise the switch between these speed modules to allow this phenomenon to be studied.

Here we used dual contralateral ventral root recordings to obtain detailed information regarding swim bout composition. We assessed and compared the fictive motor activity of three commonly used swim episode-eliciting methods to spontaneous swims. Exposure to an N-methyl-d-aspartate (NMDA) receptor agonist and electrical stimulation evoked swims with various artefacts, rendering them unsuitable as substitutes for natural swims. Swims induced through the optomotor response were most similar to spontaneous swims. A grating speed of 20 mm/s elicited swim bouts most reliably that contained both slow and fast fictive output frequencies; making this the most optimal condition to study transitions between speed modules. Subsequently, we optimized fictive locomotion in combination with calcium imaging or optogenetic stimulation of motor neurons to validate the methodological pipeline. The calcium imaging allowed us to link motor output to the activity of individual neurons, while optogenetic stimulation of motor neurons, enabled us to disrupt locomotor coordination. This strategy can be used to analyze and manipulate the activity of specific interneuron populations in the spinal cord to reveal their role in orchestrating motor output.

## RESULTS AND DISCUSSION

### Experimental setup and extraction of fictive locomotor data

**Fig. 1.**
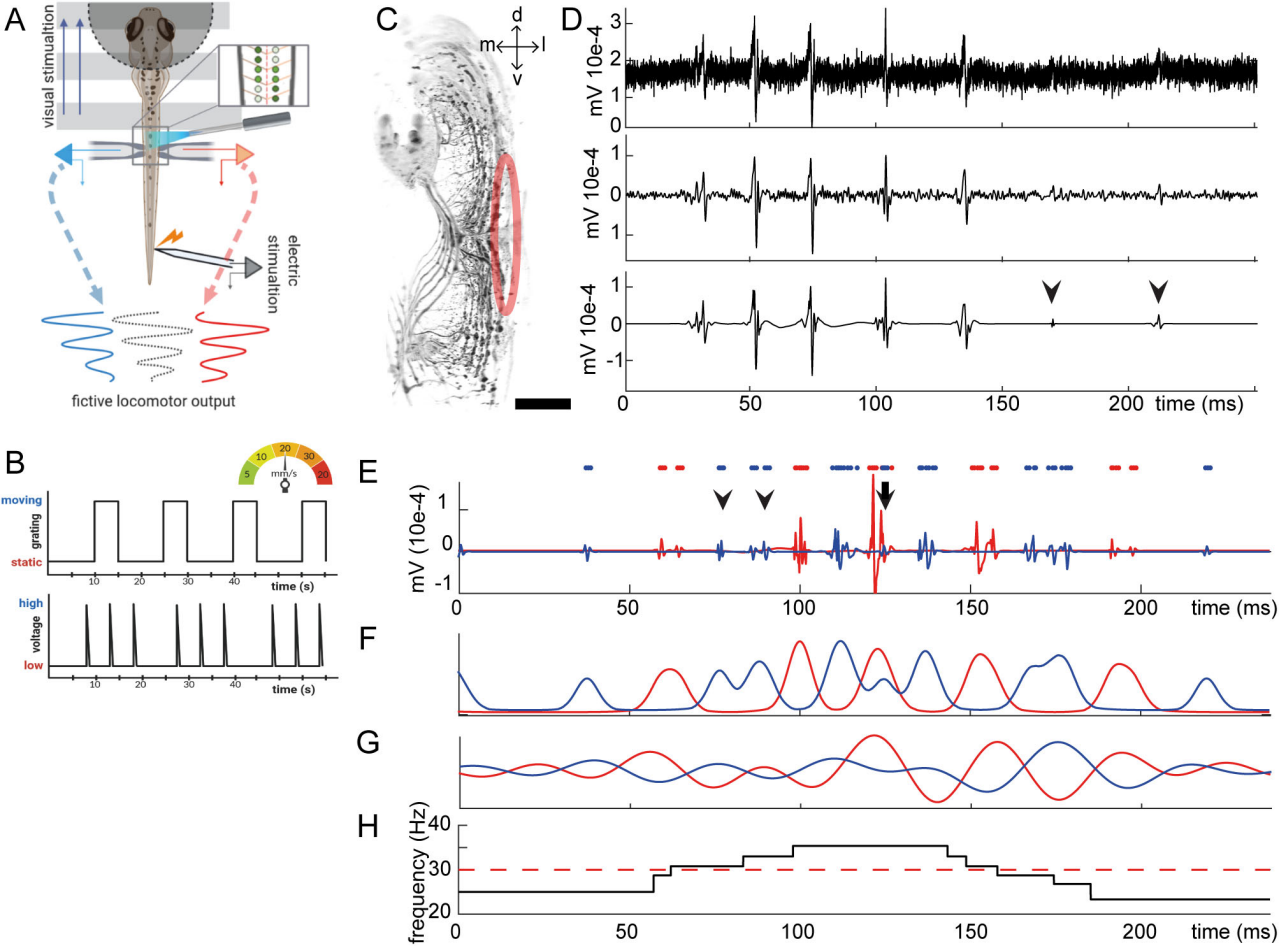
**Methodology and data analysis.** (A) Electrodes were placed on contralateral sides and stimulation electrode at the tip of the tail and an optomotor grid was projected underneath the larvae. For optogenetic neuromodulation an optic fiber was placed at the same segment as the recording electrodes. (B) Stimulation protocol for optomotor response and electric stimulation. (C) Transverse view of confocal stack of motor neurons marked by the *mnx1* promoter at 4 dpf. Red ellipse indicates approximate area of musculature covered by recording electrode. Scale bar: 15 µm. (D) Comparison of raw signal (top), conventional band-pass filtering (middle) and wavelet denoising (bottom). Arrowheads indicate spikes registered after wavelet denoising, which might be overlooked following conventional band-pass filtering. (E) Primary analysis of denoised traces with identified peaks (dots). Arrowheads indicate an instance of alternation error and arrow indicates a simultaneous burst. (F) Activity trace in which peaks correspond to burst activity and troughs to inter-burst periods. (G) Sinusoidal representation of activity trace without compromising frequency information. (H) Continuous wavelet transform produces high temporal resolution frequency output.

**Fig. 2.**
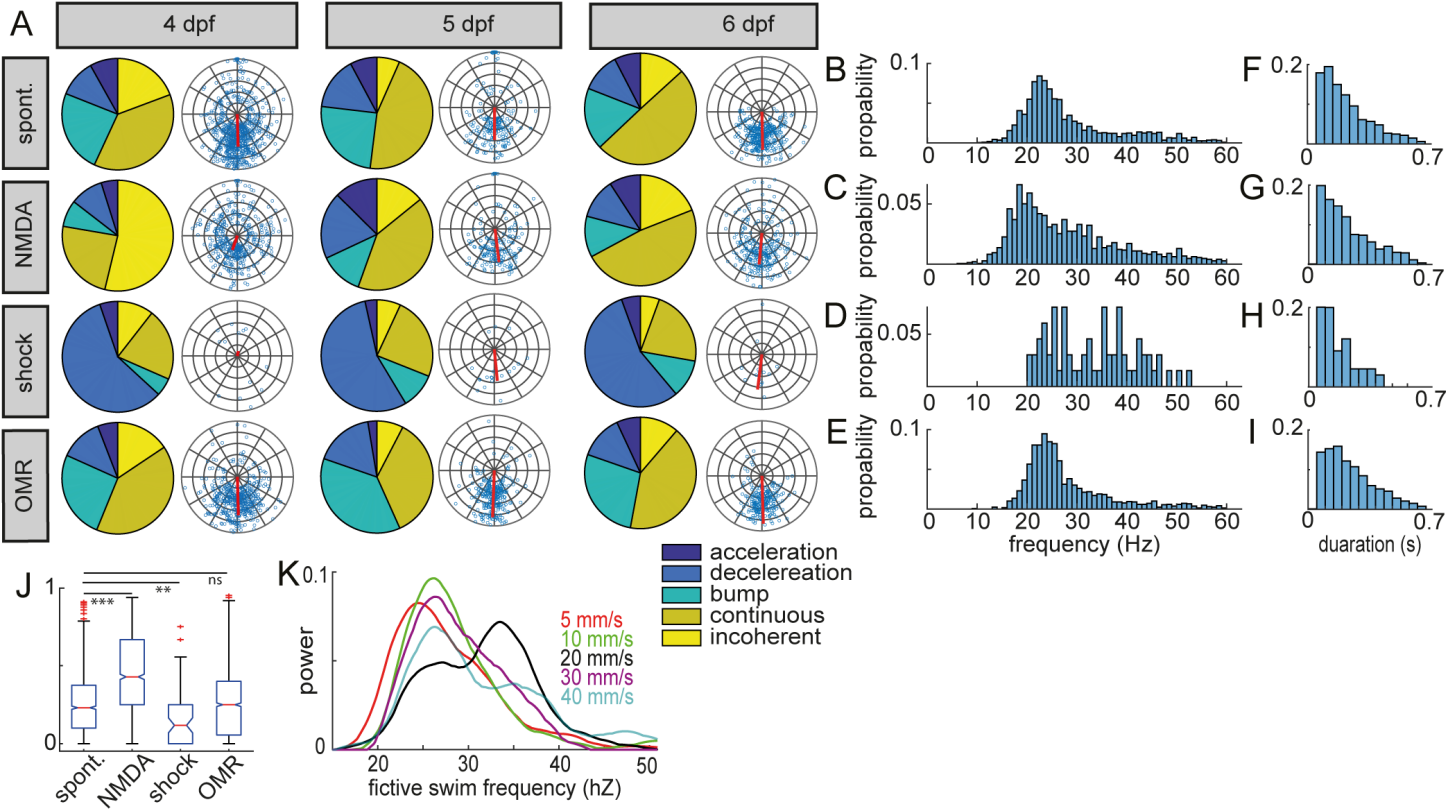
**Elicitation of fictive swims in zebrafish larvae.** (A) Spontaneous swims (4 dpf: *N*=10, *n*=724; 5 dpf: *N*=7, *n*=191; 6 dpf: *N*=7, *n*=409) compared to swims elicited through NMDA application (4 dpf: *N*=6, *n*=406; 5 dpf: *N*=3, *n*=168; 6 dpf: *N*=5, *n*=257), electric stimulation (4 dpf: *N*=3, *n*=13; 5 dpf: *N*=4, *n*=19; 6 dpf: *N*=5, *n*=14) and optomotor response (4 dpf: *N*=9, *n*=582; 5 dpf: *N*=6, *n*=273; 6 dpf: *N*=7, *n*=247). Pie charts represent distribution of swim bout types per treatment per age. Polar plots indicate phase shift and coherence between left and right hemi-segment. (B–E) Fictive locomotor output frequency distribution for spontaneous swims (B) and swim elicited by application of NMDA (C), electric stimulations (D) and optomotor response (E). (F–I) Swim duration distribution for spontaneous swims (F) and swims elicited by application of NMDA (G), electric stimulations (H) and optomotor response (I). (J) Boxplot of distribution of alternation penalty score. (K) Power frequency distribution of fictive swim output in 5 dpf larvae elicited by optomotor response at 5, 10, 20, 30 and 40 mm/s (*N*=4, 5, 3, 3 and 2, *n*=57, 76, 90, 19 and 31, respectively).

Embedded zebrafish larvae with mobile tails tend to perform spontaneous swim bouts. However, these primarily consist of slow swims (<30 Hz) (McLean et al., 2008) and occur at unpredictable time intervals (Fitzgerald et al., 2019; Palmér et al., 2017). As our interest revolves around locomotor speed changes it is crucial that a large fraction of recorded swim bouts cross the 30 Hz threshold, which marks that switch (Orger et al., 2008). Additionally, consistent elicitation of fictive locomotor activity is required for the timing of optogenetic neuromodulation, which renders the random timing of spontaneous swims unpractical. We investigated three frequently used methods for triggering swims: NMDA application, electrical stimulation to the tail and a visual stimulus to activate the optomotor response (Fig. 1A,B). Dual electrode recordings were used to measure bulk activity of the motor neuron pool enabling analysis of the alternation properties of the swim episodes (Fig. 1C). Wavelet denoising was applied to the raw data to extract electrophysiological activity with high resolution in both time and frequency domains (Fig. 1D,E). Wavelet denoising reduced background noise to a bare minimum and preserved low amplitude spikes better when compared to conventional 100–1000 Hz band-pass filtering (Fig. 1D, arrowheads). As we set out to qualitatively compare fictive output of the stimulation methods, an unbiased method of extracting swim episodes from denoised signals was required. The noiseless baseline activity resulting from wavelet denoising allowed for swim bout localization solely based on clustering of peak-finding output. No biased human interference played any role in swim episode extraction and the only exclusion criteria was episode length, where episodes lasting for less than 100 ms were excluded from analysis as this interfered with further processing. To quantify burst timing parameters and left/right alternation, an activity trace was created by applying an upper envelope filter to the denoised data, which was then filtered through a 10–40 Hz band-pass filter (Fig. 1F). Here, trace apexes correlate to burst activity and troughs to inter-burst periods. Because the band-pass filter eliminates frequencies within the larvae's output range (20–60 Hz), this activity trace was not suitable for frequency-dependent analyses. Thus, a second activity trace was constructed without any frequency based filtering to create a sinusoidal-like representation that conserves accurate frequency and phase information (Fig. 1G). This amplitude filter was used for the phase-shift analysis and extraction of time-frequency information (Fig. 1H). Phase shift analysis, as represented in polar plots (Fig. 2A), represents the lag of activity between the two recorded channels in which a phase shift of 180°, indicated by a resultant pointing downwards, perfect alternation in anti-phase; left channel is active precisely in between the previous and subsequent periods of activity in right channel and vice versa. Length of the resultant from the origin in the polar plots represent the coherence between the two signals with a large amplitude representing strong similarity between the two channels.

Two scores were applied to quantify the degree of left–right burst symmetry during swim episodes. The alternation penalty registers instances where a burst on one side is followed by another ipsilateral burst rather than the usual consecutive contralateral burst (Fig. 1E, arrowheads). The alternation penalty ranges from zero to one and was defined as the number of consecutive ipsilateral bursts divided by the total number of bursts within the fictive locomotor episode. The simultaneous burst penalty scores instances where both sides of segment display simultaneous activity (Fig. 1E, arrow) and was defined as the number of bursts overlapping by more than 70% divided by the total number of bursts during the fictive swim episode.

### Comparison of fictive swims elicited by NMDA application, electric stimulation or optomotor stimulation to spontaneous swims

Tonic application of NMDA to *in vivo* and *ex vivo* spinal cord preparations has been an historical hallmark method of eliciting fictive locomotion where the activation of NMDA receptors induces membrane potential oscillations and increased overall excitability leading fictive locomotion episodes (Alford and Williams, 1989; Grillner and Wallén, 1985; Sigvardt et al., 1985).

**Fig. 3.**
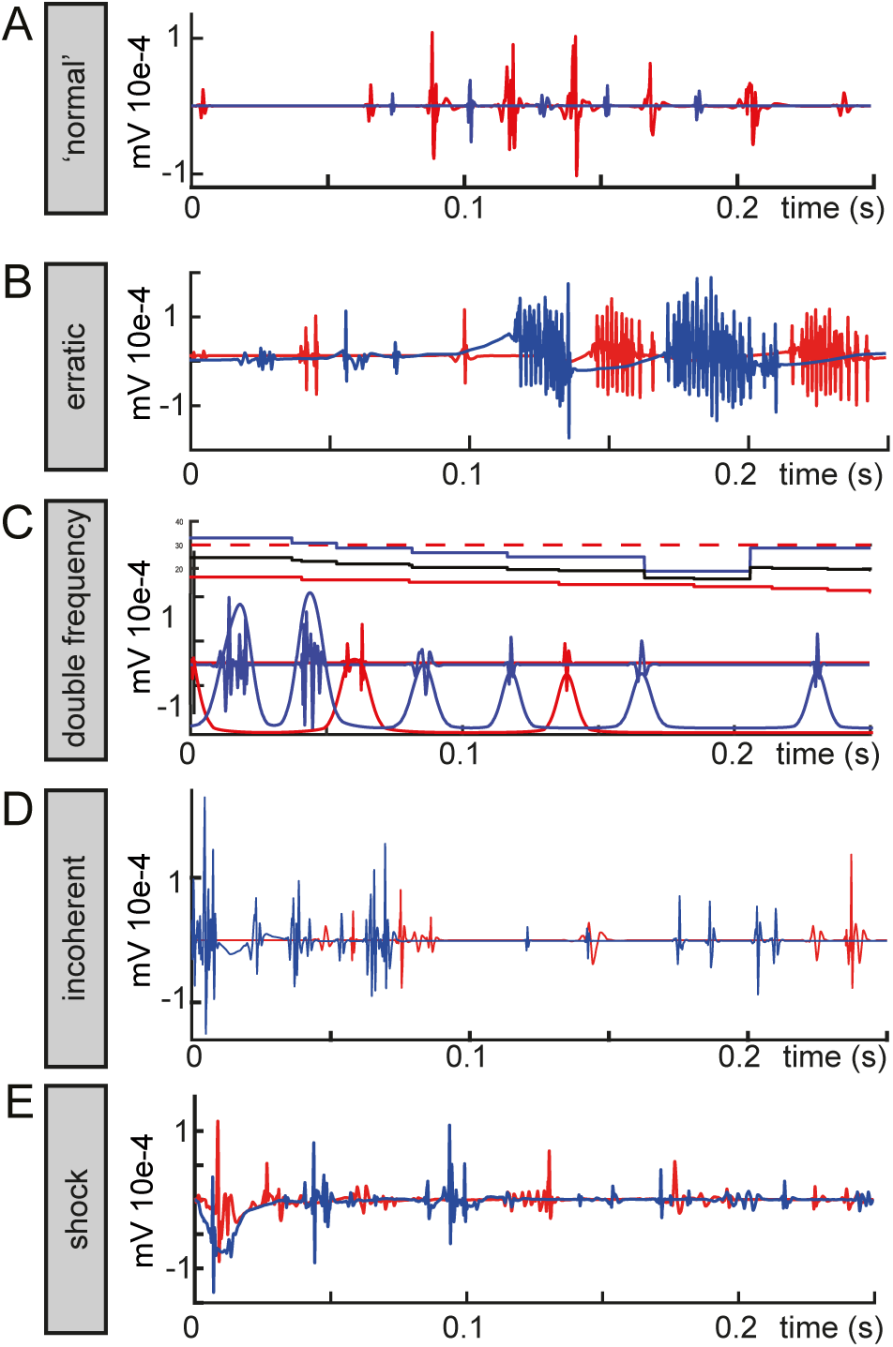
**Common artefacts during fictive swims.** (A) Example of representative spontaneous swim. (B) Erratic bursting in NMDA induced swims with an instantaneous switch from low amplitude irregular spiking to erratic bursting. (C) Discrepancy in frequency output between left and right side of the same segment induced by NMDA application. (D) Incoherent fictive locomotor activity as observed in NMDA induced swims. (E) Fictive swim output after electric stimulation displaying a stimulation artefact.

Phase-shift analysis revealed that NMDA application produced swims with lower coherence and larger phase shifts than spontaneous activity (Fig. 2A). NMDA stimulation resulted in swims with a duration similar to those of spontaneous swims (Fig. 2F,G). Application of NMDA at 4 days post fertilization (dpf) induced a disproportionate number of incoherent swims, consisting of irregular and non-rhythmic bursts and spiking (Fig. 3D), and high variation in left-right phase difference compared to spontaneous swims at that age (*P*<0.0005). Although NMDA application produced abundant swim activity, the resulting swims were shifted to a lower frequency range (<20 Hz) (Fig. 2C). NMDA induced swims produced a higher alternation penalty (0.50±0.22) and simultaneous burst penalty (0.19±0.10) than spontaneous (0.36±0.21 and 0.15±0.10, respectively) and optomotor swims (0.31±19 and 0.16±0.10) (ANOVA *P*<0.005 and *P*<0.005) (Fig. 2J). Additionally, NMDA application resulted in various fictive output artefacts. Most strikingly, an instant shift from low amplitude spiking to erratic bursting or vice versa, indicating discrepancy in locomotor network output likely due to the rise in neuronal excitability (Fig. 3B). Cases of dual frequency output were also observed where there was a large difference in the frequency output between the left and right sides (Fig. 3C). This feature would be overseen in single electrode recordings, highlighting the strength of dual electrode recording.

Application of an electric stimulus to the tail of the larva elicits an evading escape response followed by a fast swim (Budick and O'Malley, 2000; Domenici and Hale, 2019). This was evident in the mean swim frequency distribution as these swims largely lacked low frequency swims (Fig. 2D). Electrical stimulation yielded a distribution of swim types least similar to spontaneous swimming activity with a high fraction of decelerating swims with a fictive swim frequency starting above 30 Hz and then deceasing over time (Fig. 2A) and shorter swim bouts (Fig. 2H). Left–right phase difference differed significantly at 4 dpf compared to spontaneous activity (*P*<0.005). In addition to the increased complication of adding a third electrode, the electrical stimulation generated strong artefacts in the recording electrodes, preventing proper registration of the initial bursts within a swim bout (Fig. 3E). Even though electrical stimulation yielded a low alternation penalty (Fig. 2J), a higher simultaneous burst penalty (0.22±12) than spontaneous (0.15±0.10) and optomotor swims (0.16±0.10) (ANOVA *P*<0.005 and *P*<0.005) was observed. While most NMDA induced bouts did not cross the 30 Hz threshold, the case was the opposite for swims elicited through electrical stimulation, but these predominantly consisted of deceleration events (Fig. 2A).

For the optomotor response, the phase-shift analysis was strikingly similar between spontaneous and optomotor induced fictive swimming without significant difference in phase shift or coherence (Fig. 2A). At all tested ages, the optomotor response induced swimming displayed a distribution of swim types similar to those observed in spontaneous swims, with a large fraction swims that crossed the 30 Hz threshold during both acceleration and deceleration (Fig. 2A). Both mean swim frequency distribution and swim duration distribution was similar to those observed in spontaneous swims (Fig. 2E,I). Upon closer analysis of the frequency output generated by using different speeds of the grating at 5 dpf, an optimal distribution of swim frequencies was observed at a 20 mm/s grating speed (Fig. 2K). This stimulation speed produced the widest range of swim output frequencies with a peak at 26 and 34 Hz separated by a through at 30 Hz. The frequency distribution showed that the spread was widest at 5 and 20 mm/s (Fig. 2K). Although a grid speed of 10 mm/s generated a higher percentage of bouts crossing the 30 Hz threshold (43% at 10 mm/s versus 40% at 20 mm/s), this small improvement was deemed to not outweigh the robustness of triggering a swim and superior frequency distribution gained at 20 mm/s. Compared to spontaneous bouts, we found that the optomotor response elicited more swims bouts that crossed the 30 Hz threshold (19% and 25%, respectively).

Comparing the above-mentioned parameters and artefacts, swims elicited by optomotor response in 5 and 6 dpf larvae were the most similar to spontaneous swims. These results prompted us to choose an optomotor grid, moving at 20 mm/s, for the calcium imaging and optogenetic experiments.

### Calcium imaging and optogenetic stimulation of motor neurons during fictive locomotion

To display the versatility of the setup we combined the optimized stimulation method with both calcium imaging and optogenetic activation. The motor neuron population, marked by *mnx1*, was chosen as the target for calcium imaging and optogenetic neuromodulation. This allowed for a direct link between calcium activity and fictive locomotor output for verification. Similarly, as motor neurons are the final link in the locomotor networks, disturbances in motor output could be directly linked to any optogenetic neuromodulation.

**Fig. 4.**
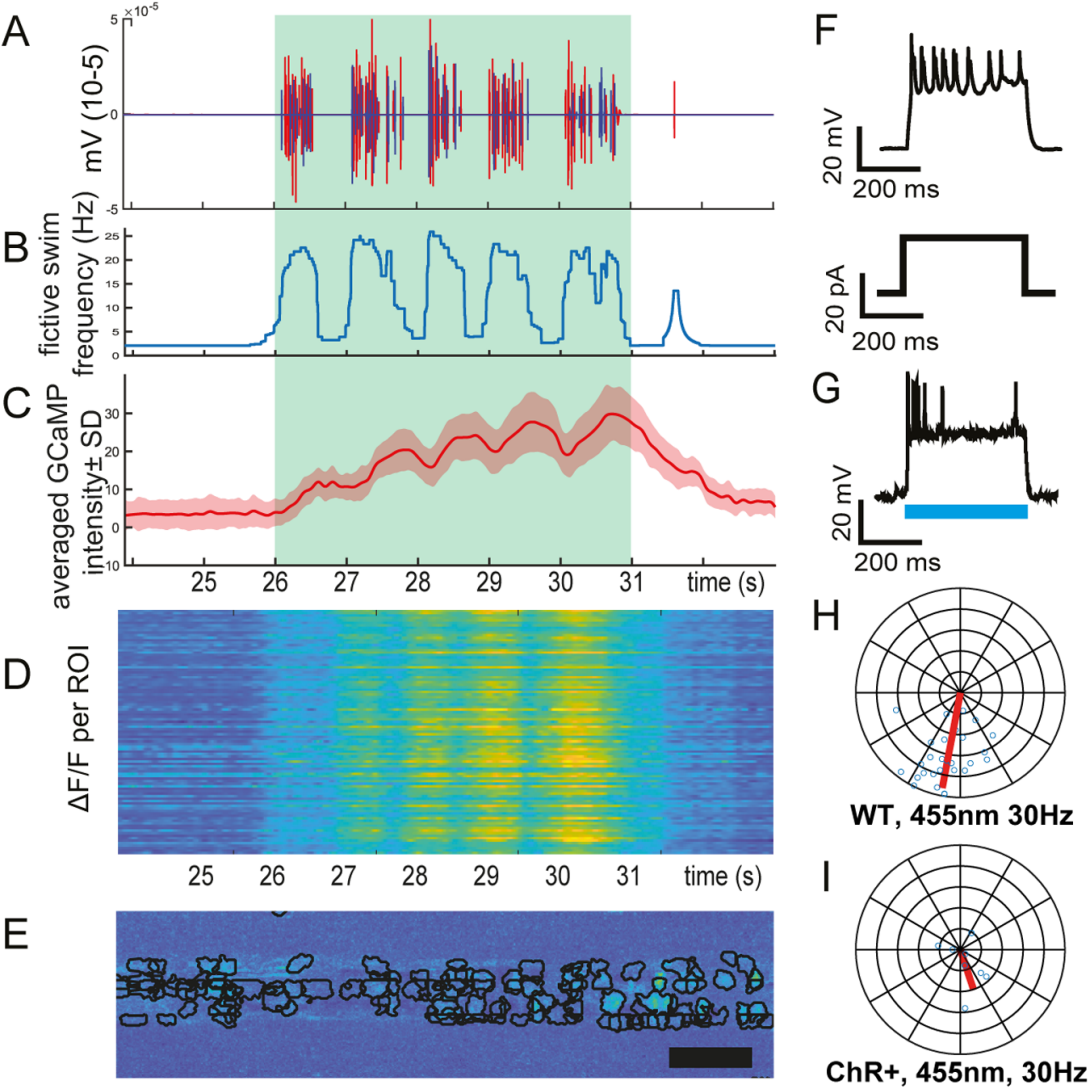
**Fictive locomotion combined with calcium imaging and optogenetic stimulation in motor neurons.** (A–E) Combined dual electrode fictive locomotion, optomotor stimulation and calcium imaging. Green box indicates the period of optomotor stimulation. Fictive locomotor output elicited by 20 mm/s optomotor stimulation (A). Fictive swim frequency corresponding (B). Δ*F*/*F* averaged over all ROIs±SD corrected for time lag between electrophysiology and calcium imaging output (C). Δ*F*/*F* transients per ROI (D). Resulting ROIs from automatic ROI segmentation as performed by CaImAn algorithm (E). (F) Current induced action potentials from Mnx-Chr2 neurons; upper trace is the induced action potentials, and the lower trace is the stimulating current pulse. (G) ChR2 activated action potentials; blue line indicates the stimulating blue light pulse. (H,I) Phase analysis of optogenetic stimulation of motor neurons during fictive locomotion in control animals (H, *N*=2, *n*=28) and tg(mnx1-Gal4; UAS-ChannelRhodopsin-mCherry) animals (I, N02, *n*=8). Scale bar: 50 µm.

General calcium dynamics and GCaMP5G decay time make it unfeasible to extract high temporal resolution information regarding firing frequencies or left–right alternation from the obtained activity data at a single cell level. However, synchronized electrophysiology and calcium recordings, driving expression of GCaMP5G in motor neurons using the *mnx1* promoter, allowed us to establish the temporal lag between GCaMP5G signal and fictive locomotion activity to 311±67 ms. This revealed that GCaMP5G is sufficiently fast to register activity of multiple sequential swim bouts with short inter-bout intervals (Fig. 4A–C). We can correlate the fictive output (Fig. 4A) to the fictive output frequency of the larvae (Fig. 4B) and visualize global calcium activity (Fig. 4C) or the traces of dozens of individual motor neurons (Fig. 4D) after automated region of interest (ROI) detection (Fig. 4E).

Optogenetic inhibition and excitation have become hallmark tools to investigate the function of neurons, both on an individual basis and at a population scale. As a proof of concept we used channelrhodopsin, expressed in motor neurons using Tg (*mnx1*:Gal4; UAS:ChR2-mCherry), to force depolarization. The performance of channelrhodopsin was validated by patch-clamp recording of individual motor neurons during stimulation with 455 nm light. Optogenetic stimulation successfully induced action potentials in the patched cells (Fig. 4F,G). We then elicited swim episodes by use of optomotor response and excited motor neurons in the segment of recording. The resulting swims displayed significantly lower coherence in animals expressing channelrhodopsin than controls (*P*<0.001), indicating that optogenetic stimulation of motor neurons significantly disturbs the fictive locomotor output (Fig. 4H,I).

### Concluding remarks

Fictive locomotion in zebrafish is an important method when interrogating the functional properties of locomotor related neuronal networks. Fictive locomotor recordings are unique in that it measures neuronal activity, which can be translated into the animal’s behavioral output. However, the effects of different swim eliciting methods and their implications on the network output and fictive behavior are seldom fully considered. Despite the diverse range of methods being used in the field to elicit fictive swims, no systematic comparison between the resulting locomotor outputs has been performed. In this study we aimed to clarify benefits and pitfalls of the different stimuli, in order to close the gap between various methodologies used in published work and provide a comparative dataset to be used when selecting stimulation protocols for future studies.

We found that swims triggered via the optomotor response at 5* *dpf were the most similar to spontaneous swims with the additional advantage that it allows for more frequent swim episodes and a higher variety in swim speeds. Both application of electric stimulation and NMDA were prone to induce technical and locomotor artefacts and are thus not suitable if ‘natural’ swims are desired. We generated a library of over 5000 swim episodes that can be used as a benchmark to compare to altered swim patterns of mutants, morphants or optogenetically neuromodulated swims. We show the possibility to combine this method of eliciting fictive swims with both calcium imaging and optogenetic neuromodulation. The activity of specific neuronal populations can be correlated to the fictive output to confirm whether the cells are rhythmically active and in phase with locomotion at different swim speeds. Changes in motor output, regarding alternation and frequency can be monitored while activating or silencing specific interneuron populations.

In all, this strategy allows a high-throughput strategy to examine novel populations identified through enhancer trap screens or via a candidate gene approach. The supplemental value of dual contralateral fictive motor recording adds a high-definition characterization of the locomotor output and enables discovery of small changes in motor coordination. Combining the approach of optomotor stimulation to reliably elicit fictive locomotion activity with the possibility to incorporate calcium imaging or optogenetic neuromodulation makes for a highly versatile tool to study the locomotor network.

## MATERIALS AND METHODS

### Fish husbandry

Transgenic zebrafish of the lines *Tg(mnx1:Gal4)* (Seredick et al., 2012), *Tg(mnx1:GCaMP5G)*, *Tg(UAS:GCaMP5G)* (Akerboom et al., 2012), *Tg(UAS:ChR-mCherry)* (Arrenberg et al., 2009) in a nacre or casper background were kept at a 14–10 light cycle at a constant temperature of 28°C. Adult fish were in-crossed to obtain larvae that were raised in darkness at 28°C until 4–6 dpf. All experiments were performed under an ethical permit obtained from a local ethical board in Uppsala, Sweden (C164/14; 14088/2019).

### Animal preparation

Larvae were screened for reporter genes at 3 dpf. Animals at experimental age (4–6 dpf) were immobilized by application of 0.04% tricaine solution (Sigma-Aldrich) and paralyzed by injecting a small bolus of the post-synaptic neuromuscular junction blocker α-Bungarotoxin (1 mg/mL, Almone Labs) into the pericardium. Tricaine was washed out by bathing the larvae in filtered tank water and paralysis occurred within 10–15 min after injection.

A single larva was placed in the lid of a 35 mm Petri dish filled with 4 mL extracellular recording solution (in mM; NaCl, 134; KCl, 2.9; CaCl_2_, 2.1; MgCl_2_, 1.2; HEPES,10; glucose,10, pH=7.8, 290–300 mOsm/mL) (Drapeau et al., 1999) and mounted ventrally by application of tissue adhesive (3 M Vetbond) to the mandibular region of the head and the tip of the tail (Fig. 1A). A seven to ten segment long stretch of skin was removed from both flanks of the larva to expose the musculature. An in-house 3D printed cover was placed over the head to shade the larvae's eyes from the epifluorescent light path while leaving the trunk and tail of the larva accessible.

### Elicitation of fictive swimming

In addition to spontaneous swimming bouts, fictive locomotion was elicited through three commonly used methods: visual stimulation to trigger the optomotor reflex, electrical stimulation and application of NMDA.

For the optomotor response, a moving grid of black and red stripes with a 12 mm period was projected onto an opaque screen covering the bottom of the preparation dish using a LED projector (Asus ZenBeam e1). The projector light passed through a 600 nm long-pass filter to avoid interference in the GCaMP5G emission and excitation spectrum. Grating speeds of 5, 10, 20, 30 and 40 mm/s were presented in a randomized manner in a protocol consisting of four repeats of 10 s of static grating followed by 5 s of moving grating (Fig. 1B).

Electric stimulation was performed by placing an in-house fabricated platinum bipolar electrode at the tip of the larvae's tail. A 10 ms electric pulse of 70–100 mA was applied through an analogue stimulus isolator (A-M Systems) in synchrony with recording equipment through a custom Matlab script. Three sets of three stimulations at 6s intervals, with a 16s interval between sets, were applied per recording (Fig. 1B).

Drug induced swimming was elicited by adding NMDA (Sigma-Aldrich) to the recording chamber at a final concentration of 100 µM in the bath (McDearmid and Drapeau, 2006; Wiggin et al., 2012). This was an intermediate working concentration based on previous fictive locomotion experiments in zebrafish (Grillner, 2006; Sigvardt et al., 1985). 2 min long recordings were taken at 2.5 min intervals for a total period of 30 min.

### Electrophysiological recordings

Mounted fish were placed in a Nikon Eclipse FN1 upright microscope. For fictive locomotion recordings, long tapered micropipettes (Harvard Instruments outer diameter 1.5 mm, inner diameter 0.86 mm) were pulled (Sutter Instruments P1000), scored and broken for a pipette tip of 50–70 µm. To accommodate smooth contact with the musculature, micropipette tips were beveled (Narishige EG-40) for a smooth tip surface and bent to a 35° angle (Narishige MF-90). Micropipettes were filled with extracellular recording solution and advanced to the musculature of contralateral sides of the same body segment. All recordings were performed at segment 10 (±5 segments). Upon contact, a small negative pressure was applied, and fictive signal appeared within 5 min. Electrophysiological signals were recorded at 50 Ks/s in current clamp mode using an Intan Technologies CLAMP amplifier.

The patch-clamp recording was used to further exam the optogenetic excitation of motor neurons. The same transgenic fish lines and screening procedure were used as described above. The prepared fish larvae (described in ‘Animal preparation’ section) was transferred to a recording chamber for electrophysiological measurements. The mCherry labelled motor neurons were identified using a Prime BSI Express Scientific CMOS camera (Teledyne Phtometrics, USA) through a 60x water-immersion objective (LUMPlan FI, NA 0.9, Olympus) using a CoolLED pE-300^white^ fluorescent LED light source. Patch electrodes (12–15 MΩ) were prepared by pulling borosilicate glass capillaries (GC150F-10 Harvard Apparatus) with a flaming micropipette puller (P-1000, Shutter Intrument, USA). Motor neurons were patched with a recording pipette filled with an internal solution containing: 130 mM K-gluconate, 40 mM HEPES, 1.02 mM MgCl2, 2.17 mM MgATP, 0.34 mM NaGTP, with pH adjusted to 7.2 using 1 M KOH. After the whole-cell patch-clamp configuration was achieved, short current pulses (500 ms) were injected to induce action potentials in current-clamp mode. To verify the effect of optogenetic stimulation, a short duration (500 ms) of a narrow band blue light (470–495 nm, filter cube U-MNIBA3, Olympus) was applied on the cell through the objective. All patch-clamp signals were amplified with a MultiClamp 700B amplifier (Axon Instruments), digitalized at 20 kHz with Digidata 1440A (Molecular Devices), low pass filtered at 10 kHz, and acquired in WinWCP software (Dr. J. Dempster, University of Strathclyde, Glasgow, UK).

### Calcium imaging

Calcium imaging was performed by epifluorescent 440 nm LED illumination (CoolLED Pe-100). Fluorescence signals of 5–50 cells were recorded at 30–100 frames per second, depending on strength of GCaMP5G expression, using a Photometrics Prime camera run in micromanager and triggered in synchrony with the stimulation protocol through Matlab. Electrophysiology, imaging and electrical/visuals stimulation were synchronously executed through a custom Matlab script. Communication between computer and hardware was accomplished through a National Instruments PCI-6221 DAQ and BNC2110 breakout board.

### Optogenetic stimulation

For optogenetic excitation of motor neuron activity, adult transgenic fish of the *Tg(mnx1:*Gal4*)* promotor line were crossed with a *Tg(UAS:ChR-mCherry*) driver line. Larvae were screened at 3 dpf for strong mCherry expression and prepared as described in the ‘Animal preparation’ section. Additionally, an optic fiber (50 µm diameter) was placed at 50 µm from the spinal cord, illuminating the spinal cord with 455 nm (1 W/cm^2^) at a slight angle rostral of the recording electrodes using a Prizmatix mix-LED. Illumination covered two to three body segments. Larvae were presented with the same optomotor protocol as described above. During second moving grid period no optogenetic stimulation was applied to serve as control. For the remaining optomotor periods, optogenetic activation was applied during the third and fourth second of the five-second optomotor period.

### Data analysis

The calcium imaging data was run through the CaImAn algorithm (Giovannucci et al., 2019) for automated ROI detection and signal deconvolution. Inferred calcium activity traces were then further analyzed with the electrophysiology data in a custom written Matlab script.

Fictive locomotion recordings were denoised by application of continuous wavelet transform utilizing a Morlet wavelet. Peak-finding was applied to the denoised traces to find all spikes in the traces, the temporal locations of the found peaks were then clustered to define start and end points of swimming epochs used to extract the periods of fictive locomotor activity from each trace (Fig. 1D,E). An amplitude filter was fitted to the trace and smoothened, resulting in a periodicity trace per electrode, which is high (∼1) during neural activity and low (∼0) in periods of no activity. These traces were used to extract time-frequency and phase difference information. The resulting library of activity epochs was then further analyzed based on stimulation method, swim frequency and frequency pattern over time within the swim epoch; increasing frequency, decreasing frequency, continuous frequency or a rise and then fall in frequency. Fictive swim episodes were broken down further by clustering spike times into activity bursts. The activity bursts within each fictive swim episode were quantified into burst duration, frequency and spike number (Fig. 1E). To quantify and compare the precision of left-right alternation within and between bouts, an alternation penalty and simultaneous burst penalty was used. All signal processing and analysis scripts are available upon request to the corresponding author.
